# Blood Metabolomic Profiling Confirms and Identifies Biomarkers of Food Intake

**DOI:** 10.3390/metabo10110468

**Published:** 2020-11-17

**Authors:** Julia Langenau, Kolade Oluwagbemigun, Christian Brachem, Wolfgang Lieb, Romina di Giuseppe, Anna Artati, Gabi Kastenmüller, Leonie Weinhold, Matthias Schmid, Ute Nöthlings

**Affiliations:** 1 Unit of Nutritional Epidemiology, Department of Nutrition and Food Sciences, Rheinische Friedrich-Wilhelms-University Bonn, 53115 Bonn, Germany; koluwagb@uni-bonn.de (K.O.); cbrachem@uni-bonn.de (C.B.); noethlings@uni-bonn.de (U.N.); 2 Institute of Epidemiology, Kiel University, 24105 Kiel, Germany; wolfgang.lieb@epi.uni-kiel.de (W.L.); r.digiuseppe@kompetenznetz-darmerkrankungen.de (R.d.G.); 3 Kompetenznetz Darmerkrankungen e.V., 24103 Kiel, Germany; 4 Research Unit Molecular Endocrinology and Metabolism, Helmholtz Zentrum München, German Research Center for Environmental Health (GmbH), 85764 Munich, Germany; anna.artati@helmholtz-muenchen.de; 5 Institute of Computational Biology, Helmholtz Zentrum München, German Research Center for Environmental Health (GmbH), 85764 Munich, Germany; g.kastenmueller@helmholtz-muenchen.de; 6 Institute for Medical Biometry, Informatics and Epidemiology (IMBIE), University Hospital Bonn, 53127 Bonn, Germany; weinhold@imbie.meb.uni-bonn.de (L.W.); matthias.schmid@imbie.uni-bonn.de (M.S.)

**Keywords:** untargeted metabolomics, biomarkers, dietary intake, metabolites, dietary assessment methods

## Abstract

Metabolomics can be a tool to identify dietary biomarkers. However, reported food-metabolite associations have been inconsistent, and there is a need to explore further associations. Our aims were to confirm previously reported food-metabolite associations and to identify novel food-metabolite associations. We conducted a cross-sectional analysis of data from 849 participants (57% men) of the PopGen cohort. Dietary intake was obtained using FFQ and serum metabolites were profiled by an untargeted metabolomics approach. We conducted a systematic literature search to identify previously reported food-metabolite associations and analyzed these associations using linear regression. To identify potential novel food-metabolite associations, datasets were split into training and test datasets and linear regression models were fitted to the training datasets. Significant food-metabolite associations were evaluated in the test datasets. Models were adjusted for covariates. In the literature, we identified 82 food-metabolite associations. Of these, 44 associations were testable in our data and confirmed associations of coffee with 12 metabolites, of fish with five, of chocolate with two, of alcohol with four, and of butter, poultry and wine with one metabolite each. We did not identify novel food-metabolite associations; however, some associations were sex-specific. Potential use of some metabolites as biomarkers should consider sex differences in metabolism.

## 1. Introduction

Dietary intake can be assessed with various methods, all of which have strengths and limitations. Self-reported methods, such as questionnaires, are relatively easy to implement, but reporting bias and measurement error are major challenges [1,2]. Thus, suboptimal estimation of actual dietary intake and inconsistent findings for diet-disease associations might be due to measurement errors [3]. In general, biomarkers are considered more objective compared to self-report methods.

The traditional approach in development of dietary intake biomarkers is to measure one or more hypothesis-driven biomarkers at a time. However, recent advances in omic analytical techniques, specifically metabolomics, have offered a data-driven approach for the development of dietary intake biomarkers that allows the assessment of many potential biomarkers at the same time [4]. Metabolomics-based biomarkers of dietary intake could overcome some of the limitations of measuring dietary intake by self-reports, and they could be an alternative or supplement to traditional dietary assessment methods [5]. A prominent example for a metabolomic-based biomarker is proline betaine, which was observed in different observational studies [6,7,8,9] to be associated with citrus fruit intake. 

An attempt has been made to develop a validation process for such newly derived dietary biomarkers [10]. The goal of validation is to ensure that potential biomarkers can reliably and reproducibly predict dietary intake. An important step in the validation of dietary biomarkers is the replication of such associations in independent samples [10]. However, the replication of food-metabolite associations is challenging. This is due to heterogeneity across study populations. This heterogeneity includes the inevitable biological variation across populations, differences in dietary assessments instruments, biosample collection and processing, technical variation in metabolomics profiling and instruments, metabolomics data-preprocessing, and statistical approaches for exploring food-metabolite associations [11,12]. Associations that are observed across populations independent of these differences may indeed be worthwhile and may serve as a first step in their validation. Thus, obtaining replicable food-metabolite associations across multiple observational epidemiological studies is an important step in the potential identification of a particular metabolite as a dietary biomarker. Due to the complexity of the metabolome, a food-metabolite association may be considered worthy of being replicable if the association has been observed in two or more independent studies. 

Furthermore, since the research field of identifying metabolomics-based biomarkers for dietary intake is relatively new, any information from the metabolome as it relates to dietary intake might be valuable. Previous studies [13,14] have shown sex-specific differences in energy and macronutrient metabolism resulting in distinct metabolic profiles, suggesting that it might be worthwhile to explore sex-specific relationships of foods with metabolites. 

In the present analyses, we were thus aiming (1) to confirm previously reported food-metabolite associations and (2) to identify novel potential metabolomics-based biomarkers of food group intake separated by sex, using an untargeted metabolomics approach.

## 2. Results

### 2.1. Characteristics of the Study Population

The characteristics of the study population are presented in Table 1. The median age of study participants was approximately 62 years. Men had a slightly higher BMI, higher waist circumference, and were more likely to be more educated and full-time employed than women. Women were more physically active compared to men. Hypertension, CHD, and diabetes were more prevalent in men. Approximately 15% of men and women were current smokers. Total energy intake and the intake of alcohol, butter, coffee, fish, mushrooms, and red meat were higher in men compared to women. The usual dietary intake of all foods used in the present study is shown in Supplemental Table S1. 

### 2.2. Results of the Systematic Literature Search

**Figure 1:**
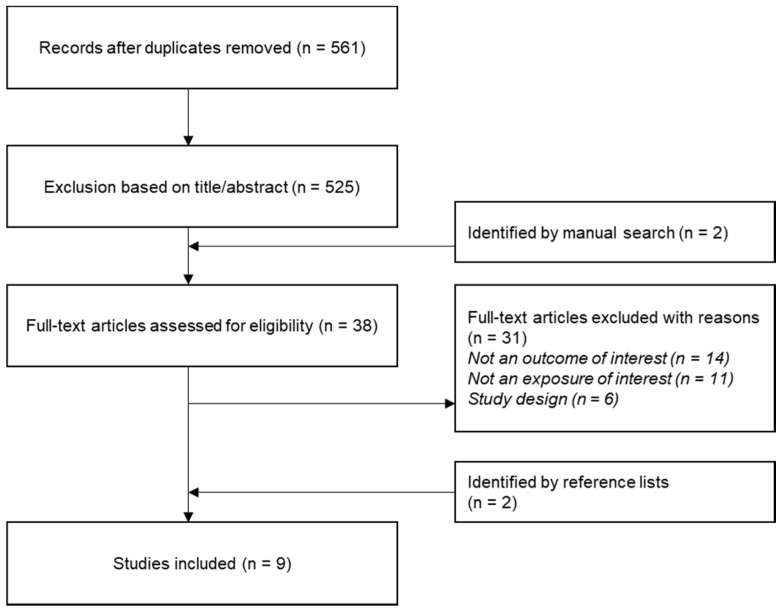
Flow chart describing the selection process in the systematic literature search.

In Figure 1 the results of the literature search for food-metabolite associations are shown. Overall, 561 articles were identified after removing duplicates. A total of 525 articles were excluded after reading the title and abstract. Two additional references were identified by manual search. Of 38 articles, 31 were excluded after reading the full text due to various reasons, such as no outcome or exposure of interest, or no suitable study. Two references were included based on reference lists of other studies. Finally, nine studies were included in the present study (Table 2).

### 2.3. Replication of Food-Metabolite Associations

The food-metabolite associations identified in our systematic review are shown in Table 2. Details of these studies can be found in Supplementary Table S2. In total, there were 82 associations reported in at least two independent study populations. These 82 food-metabolite associations comprise 22 food groups. Out of these, ten food groups (alcohol, butter, chocolate, coffee, fish, liquor, mushrooms, poultry, red meat, and wine) were assessed in our study. The remaining 12 food groups identified in our systematic review were not assessed in the present study due to the lack of coverage of both food items/groups and metabolites (“apples and pears”, “juices”), metabolites (“milk”, “tea”), food groups/items (“decaffeinated coffee”, “fish and seafood”, “soymilk”, “peanuts”, “shellfish”, “fruit juices”), or due to highly heterogeneous definition across the studies (“meat”, “nuts”). Thirty-three of the metabolites assessed in our study were associated with only one food group. Further, five metabolites assessed in our study were associated with more than one food group. In detail, the metabolite pyroglutamine was associated with two food groups (poultry and red meat), 3-carboxy-4-methyl-5-propyl-2-furanpropionate (CMPF) was associated with three food groups (alcohol, fish, and wine), ergothioneine was associated with two food groups (mushrooms and alcohol), and the metabolites 5α-androstan-3β, 17β-diol disulfate, and α-hydroxyisovalerate were each associated with two food groups (liquor and alcohol). In total, 44 of the 82 reported associations were assessed in the present study using multivariable models. We were able to confirm (*p*-value ≤ 0.05) 26 out of these 44 associations (Supplemental Table S3). 

**Figure 2:**
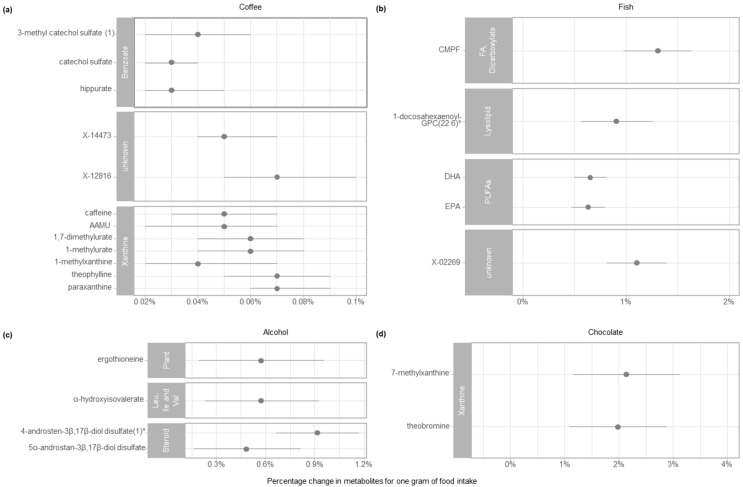
Confirmed food-metabolite associations for the food groups: (**a**) Coffee, (**b**) fish, (**c**) chocolate, and (**d**) wine. The figure represents the percentage change in metabolites for one gram change of (**a**) coffee, (**b**) fish, (**c**) alcohol, and (**d**) chocolate, respectively, and the subpathways of the metabolites; the estimates in percentage were generated from linear regression models with natural log-transformed metabolites as the dependent variables and (**a**) coffee, (**b**) fish, (**c**) alcohol, and (**d**) chocolate as the independent variable. * No pure compound was available, but we are confident in its identity. Abbreviations: AAMU, 5-acetylamino-6-amino-3-methyluracil; CMPF, 3-carboxy-4-methyl-5-propyl-2-furanpropionate; FA, fatty acid; DHA, docosahexaenoic acid; EPA, eicosapentaenoic acid; GPC, glycerophosphochcline; Leu, Ile, and Val, Leucine, isoleucine and valine metabolism; PUFAs (n-3 and n-6), polyunsaturated fatty acid (n-3 and n-6).

Overall, of the ten food groups for which we conducted replication analyses, we were able to confirm associations for coffee, fish, alcohol, chocolate, butter, poultry, and wine. Figure 2 shows the confirmed food-metabolite associations for the food groups (coffee, fish, alcohol, and chocolate) for which we have confirmed more than one association. The confirmed associations for the food groups butter, poultry, and wine are shown in Table 3.

A 1-g increase in coffee intake per day was associated with an increase of the following benzoate metabolites: 3-methylcatechol sulfate (1) (0.04%), hippurate (0.03%), and catechol sulfate (0.03%). Additionally, a 1-g intake of coffee per day was associated with an increase of the unknown metabolites X-14473 and X-12816 by 0.06% and 0.07%, respectively. A 1-g intake of coffee per day was associated with an increase of the xanthine metabolites: caffeine (0.05%), 5-acetylamino-6-amino-3-methyluracil (AAMU, 0.05%), 1,7-dimethylurate (0.06%), 1-methylurate (0.06%), 1-methylxanthine (0.05%), theophylline (0.07%), and paraxanthine (0.07%). Further, significant interaction of sex with coffee for X-14473 existed. We were not able to confirm the associations of coffee with 1,3,7-trimethylurate, cyclo(leu-pro), N-(2-furoyl)glycine, X-17185, 3-hydroxyhippurate, cinnamoylglycine, and 3-(3-hydroxyphenyl)propionate. A 1-g intake of fish per day was associated with an increase in 3-carboxy-4-methyl-5-propyl-2-furanpropionate (CMPF) by 1.3%, 1 docosahexaenoyl GPC (22:6) by 0.91%, DHA by 0.65%, EPA by 0.62%, and X-02269 by 1.11%. Further, a 1-g intake of alcohol per day was associated with an increase in ergothioneine by 0.5%, α-hydroxyisovalerate by 0.58%, 5α-androstan-3β,17β-diol disulfate by 0.48%, and 4-androsten-3β,17β-diol disulfate 1 * by 0.92%. We were not able to confirm the association between alcohol and CMPF. A 1-g intake of chocolate per day was associated with an increase in 7-methylxanthine by 2.12% and theobromine by 1.99%. Additionally, an increase in wine intake of 1 g per day was associated with an increase in X-11795 by 0.04%. We were not able to confirm the association for wine with piperine and CMPF. A 1-g intake of butter per day was associated with an increase in 10-undecenoate (11:1n1) by 0.31%, however, we were not able to confirm the association for butter and caprate 10:0. Additionally, 1-g intake of poultry per day was associated with an increase in 3-methylhistidine by 1.08%, however, we were not able to confirm the association with pyroglutamine. The confirmed food-metabolite associations and their corresponding (back-transformed) beta coefficients, confidence intervals, and *p*-values are presented in Supplemental Table S3. We were not able to confirm the associations between mushrooms and ergothioneine, poultry and pyroglutamine, red meat with pyroglutamine and X-11381 and liquor with α-hydroxyisovalerate and 5α-androstan-3β, 17β-diol disulfate.

### 2.4. Identification of Novel Food-Metabolite Associations

In the training datasets, we observed 114 and 57 significant (*p*-value ≤ 0.05) food-metabolite associations in women and men, respectively (Supplemental Tables S4 and S5). These associations were used to generate hypotheses about potential biomarkers, which were evaluated in the test dataset. We did not identify novel food-metabolite associations; however, we found food-metabolite associations in the test data that have been reported in previous studies. In our study, these associations were sex-specific. In detail, in women coffee was associated with the unknown metabolite X-14473 (1-g intake per day was associated with an increase by 0.13%), and in men coffee was associated with paraxanthine (1-g intake per was associated with an increase by 0.08%). Further, in men, a 1-g intake of fish per day was associated with an increase in EPA by 0.68% and the unknown metabolite X-02269 by 1.12%. Table 4 shows the food-metabolite associations that were confirmed (*p* ≤ 0.05) in the test data. The back-transformed beta coefficients are presented in Supplemental Table S6.

## 3. Discussion

The present study aimed to confirm blood metabolite-food associations reported in previous studies and to identify novel metabolomics-based biomarkers of food group intake in a general population sample from Northern Germany. Overall, we were able to confirm 26 out of 82 previously reported associations. Specifically, we confirmed the association of coffee with 12 metabolites, of fish with five metabolites, of chocolate with two metabolites and of alcohol, butter, poultry, and wine with one metabolite each. 

We did not identify novel food-metabolite associations; however, we confirmed food-metabolite associations in the test data that have been reported in previous studies. The confirmed associations were sex-specific; in women coffee was associated with the unknown metabolite X-14473. In men coffee was associated with paraxanthine, and fish was associated with EPA and the unknown metabolite X-02269.

### 3.1. Associations of Metabolites with Coffee

Most (*n* = 28) of the food-metabolite associations in literature were found for coffee [8,9,16,18,19]. Of these 28 metabolites, we detected 19 metabolites in our sample and confirmed associations with 12 different metabolites (catechol sulfate, 3-methylcatechol sulfate (1), X-12816, X-14473, paraxanthine, theophylline, 1-methylxanthine, 1-methylurate, 1,7-dimethylurate, AAMU, caffeine, and hippurate). All the above-mentioned studies have found associations in a positive direction. Our study is consistent with these findings. Caffeine is a purine alkaloid that occurs naturally in coffee beans and its metabolism is well explored [20]. Overall, we confirmed six caffeine metabolites (paraxanthine, theophylline, 1-methylxanthine, 1-methylurate, 1,7-dimethylurate, AAMU) which are positively associated with coffee intake. Three of the confirmed metabolites (catechol sulfate, 3-methylcatechol sulfate (1), hippurate) belong to benzoate metabolism, which is naturally occurring in coffee, and are positively associated with coffee intake [18]. Catechol, a derivative of coffee processing, is conjugated to sulfate in plasma [18,21]. Hippurate is an acyl glycine of endogenous origin and a normal component of urine [20]. It is increased with increased intake of phenolic compounds such as tea, wine and fruit juices [20]. Further, it is found after the consumption of whole grain [20]. Additionally, we confirmed associations with two unknown metabolites (X-12816, X-14473). Since they are without biochemical identities, it is difficult to provide explanations. However, updated metabolomic platforms may identify the unknown metabolites.

A three-stage clinical trial by Cornelis et al. [22] that aimed to identify individual metabolite changes in response to different coffee exposures over three months, found 82 known (and 33 unknown) metabolites that changed with coffee consumption. Observational studies found significantly fewer metabolites associated with habitual coffee consumption; however, these studies have reported novel metabolites associated with coffee. One reason could be that associations explored in clinical trials are sensitive but not specific enough to a particular food as other potential food sources of these metabolites are not considered during the intervention [23]. According to the authors, another reason could be that the duration of the study was not sufficient to picture the habitual coffee consumption observed in observational studies. In general, more controlled/intervention studies are needed that compare their results (short-term dietary intake) with results of habitual dietary intake of observational studies. 

### 3.2. Association of Metabolites with Fish Intake

Fish intake is consistently associated with five metabolites (CMPF, 1-docosahexaenoyl-GPC (22:6) *, EPA, DHA, X-02269) [7,8,15,16]. All metabolites were measured in our study, and we confirmed their associations with fish intake. Similar to the previous studies, we found positive associations for the aforementioned fish metabolite associations. CMPF is considered to be a potent uremic toxin [24] and it is assumed to be formed by the consumption of fish, vegetables, and fruits [20]. EPA and DHA are omega-3 (n-3) long-chain polyunsaturated fatty acids (n-3 LCPUFAs) which are fish biomarkers with a high degree of specificity to fish and shellfish [25]. It is suggested that erythrocyte membranes and adipose tissue are more reflective of habitual fish intake [26]. The fact that we found association of habitual fish intake and serum EPA and DHA indicates that the serum also reflects habitual fish intake. Additionally, we found an association with an unknown metabolite (X-02269). As discussed before, it is difficult to admit explanations for this metabolite without knowing its chemical structure.

### 3.3. Association of Serum Metabolites with Chocolate Intake

Chocolate has been shown to be positively associated with two metabolites (7-methylxanthine, theobromine) [6,7,16]. We measured the two associated metabolites and confirmed these associations. Theobromine is a bitter alkaloid found in the cacao tree and other plants. It is the primary alkaloid found in cocoa and chocolate [20] and it also is a caffeine metabolite [18]. 7-methylxanthine is a purine component originating from the metabolism of methylxanthines which includes also theobromine [20]. The fact that the present study confirms these associations, found at least twice in previous studies, suggests that these metabolites could be considered potential biomarkers of chocolate intake. However, as suggested by Michielsen and colleagues, it is possible that cocoa (product) specific biomarkers are not available because some of the potential biomarkers have also been found in foods with a similar composition (e.g. caffeine in coffee). [27]. Interestingly, these authors proposed that a combination of metabolites might help to discriminate profiles between cocoa (products) and foods with similar composition.

### 3.4. Association of Serum Metabolites with Wine Intake

There were consistent associations of wine with seven metabolites (scyllo-inositol, X-01911, X-11795, piperine, ethyl glucuronide, CMPF, 2,3-dihydroxyisovalerate) [6,7,15,16]. The metabolites X-01911, X-11795, piperine, and CMPF were measured in our study and we confirmed the association for unknown metabolite X-11795. In agreement with the previous studies, we found a positive association for this metabolite. 

### 3.5. Association of Serum Metabolites with Alcohol Intake

Alcohol consumption is consistently associated with eight metabolites (ethyl glucuronide, 5-α-androstan-3β,17β-diol disulfate, 4-androsten-3β, 17β-diol disulfate 1 *, α-hydroxyisovalerate, ergothioneine, CMPF, 2,3-dihydroxyisovalerate, 5α-androstan-3α,17β-diol disulfate) [7,8,15,16]. We measured ergothioneine, α-hydroxyisovalerate, 5α-androstan-3β, 17β-diol disulfate, 4-androsten-3β, 17β-diol disulfate 1 *, and CMPF in our study and found significant associations for all, except CMPF. All of the aforementioned studies have found associations in a positive direction. Our study is consistent with these findings. Ergothioneine is a metabolite of histidine and is biosynthesized by fungi and mycobacteria [20]. The highest levels of ergothioneine have been detected in mushrooms [20]. Since ergothioneine has strong antioxidant properties, it is used as a preservative in various foods, including wine [28,29]. The metabolites 5α-androstan-3α,17β-diol disulfate, and 4-androsten-3β, 17β-diol disulfate 1 * belong to the class of sulfated steroids [20]. One pathway by which the production of steroid hormones can be controlled is by the hypothalamic-pituitary-adrenal (HPA) axis [30]. Alcohol stimulates the HPA axis, which might explains the positive association between alcohol and these two metabolites [31].

### 3.6. Association of Serum Metabolites with Butter Intake

There were consistent associations of butter with four metabolites (caprate (10:0), 15-methylpalmitate (isobar with 2-methylpalmitate), 10-undecenoate (11:1n1), pentadecanoate (15:0)) [6,7,15,16]. The metabolites caprate and 10-undecenoate (11:1n1) were measured in our study and we confirmed the association for 10-undecenoate (11:1n1). In agreement with previous studies we found a positive association with this metabolite. However, the connection with butter intake remains unclear.

### 3.7. Association of Serum Metabolites with Poultry Intake

Poultry intake has been reported to be positively associated with 3-methylhistidine [15,16], as also observed in the present study. 3-methylhistidine belongs to the class of histidine and derivatives and is known as a meat-related biomarker, in particular for chicken [20]. 

### 3.8. Food-Metabolite Associations Not Confirmed in This Study

We considered studies whose food groups corresponded to our food groups. However, it is possible that even small differences in the definition of food groups across the studies lead to the fact that we could not replicate all previously reported food-metabolite associations. Nevertheless, as most of the previously reported food-metabolite associations were replicated in the present study, the likelihood of this is small. In addition, it is possible that our analysis may not have been powered enough to detect all previously reported food-metabolite associations. It is also possible that food-metabolite associations were influenced by variations in the metabolites and that in our study sample we could only detect associations with metabolites that showed higher variation.

### 3.9. Identification of Novel Food-Metabolite Associations

Finally, we did not identify new food-metabolite associations. However, we confirmed associations of fish with the unknown metabolite X-02269 and EPA and for coffee with paraxanthine in men. Further, we confirmed associations of coffee with the unknown metabolite X-14473 in women. The fact that there were distinct food-metabolite associations for each sex suggests that the metabolism of these foods could be modulated by sex hormones [13]. This indicates that the proportion or percent change in some metabolites due to specific foods vary between men and women. The significant interaction of sex for X-14473 in our replication analysis also partially confirm this sex-specificity. To substantiate that no food-metabolite associations was shared by both sexes, we performed this analysis for the whole study population adjusted for sex and sex-food interaction. We found associations of coffee with paraxanthine and theophylline, and fish with DHA. This finding suggests that our sex-specific analysis may not have been powered enough to detect the association of coffee with paraxanthine in women, as well as coffee with theophylline and fish with DHA in both sexes.

### 3.10. Further Aspects of the Study

In order to confirm previous food-metabolite associations, we considered studies whose food groups corresponded to our food groups. The defined food groups were in part highly heterogeneous across the studies, which limited the number of associations analyzed. Nevertheless, it cannot be excluded that factors such as cooking methods or subtypes of food might have influenced food-metabolite associations, as previously shown for seafood [32]. Interestingly, most of the food-metabolite associations were found for coffee intake. A possible explanation for this could be that coffee is a relative homogenous food group, although bean type, roast, and preparation methods for coffee may also play an important part [19]. Another possible reason could be an influence of the time at which the samples were taken. We investigated samples of fasting blood that are usually taken in the morning. In addition to water consumption, coffee is also allowed before the blood is taken. For this reason, and because people consume coffee especially in the morning, it is likely that the participants drank coffee immediately before the blood sample was taken.

A reliable dietary biomarker should be exposure specific [33]. For some of the confirmed metabolites research indicates that they are not exposure specific, such as caffeine in coffee and theobromine in chocolate which can also be found in other foods [27]. Hence, identification of reliable dietary biomarkers is a challenge that can only be overcome with further research. A broader metabolomics profiling and using a combination of multiple metabolites as (composite) biomarkers may help to expand the knowledge in this field.

### 3.11. Strengths and Limitations

A strength of this study is that we confirmed food-metabolite associations, which have been identified in at least two independent observational studies to establish clear and consistent associations between dietary intake and metabolites. Using an untargeted metabolomics approach, the present study provides analysis for a broad spectrum of metabolites from different metabolic pathways, as well as xenobiotics, and enables the identification of novel potential dietary biomarkers. Additionally, we aimed to identify novel food-metabolite associations conducting sex-specific analyses a priori. Additionally, we validated our results internally by splitting the sex-stratified datasets equally (1:1) into training and test datasets. We used serum samples in a relatively large study population to investigate sex-specific food-metabolite associations with an untargeted metabolomics approach. Finally, we adjusted the analyses for a comprehensive set of covariates and for multiple testing by applying the Bonferroni correction method, notwithstanding multiple significant food-metabolite associations were detected. 

A limitation of the present study is that the sample comprises adult participants from Northern Germany which limits the generalizability of the observed findings. Further, habitual dietary intake was assessed at one single time point by an FFQ. Indeed, habitual dietary intake may be better captured using a combination of dietary instruments, such as multiple 24 h dietary recalls (24HR), alone or in combination with FFQ [34]. In general, biases may occur due to residual or unmeasured confounding. In the present study, we focused on the measurement of blood metabolites by an untargeted metabolomics approach. However, not all of the potential biomarkers identified previously were measured in our study. For example, studies indicate that the metabolite trigonelline, which was not measured in our study, seems to be a promising biomarker for coffee as it is strongly correlated to coffee intake [18,19,35] and its presence in food is also largely limited to coffee [19]. This shows how important updated platforms are for identifying potential biomarkers. Further, metabolites were measured at one single time point. This one-time measurement of metabolites could be exposed to short-term variation and may not represent habitual dietary intake. Thus, day-to-day variation in metabolite levels could lead to biases in food-metabolite associations. In general, technical variation and data pre-processing may introduce biases in metabolomics. However, the fact that we were able to replicate a considerable number of consistent associations suggest that these factors have minimal impact on the food-metabolite associations that we reported in this study. As shown by others [36], the complexity of jointly consumed foods and their relationship with correlated metabolites indicates that exploring the relationship of patterns of intake of food groups and metabolite patterns may provide important insights into the relationship between food intake and metabolites. Further, future studies should consider sex-specific differences in metabolism by exploring sex-specific relationships of foods with metabolites. In addition, future studies should aim to standardize sample collection, analysis and metabolomics analysis protocol, data-preprocessing, and downstream analysis. This may not be doable in practice. There are several projects that aim to integrate metabolomics datasets. Researchers should harness these extensive data for future projects. Large-scale multicenter studies that have consistent implementation of these standardized approaches would also be good.

## 4. Subjects and Methods

### 4.1. Study Design and Population

Between 2005 and 2007, 1316 individuals of the general population in the German town of Kiel were recruited by the PopGen biobank mainly as a reference sample for genetic analyses (PopGen control sample) [37]. Medical history, lifestyle, and food intake were assessed using questionnaires. Details on study design and conduct have been described elsewhere [37,38]. For the present cross-sectional analysis, data from the first follow-up were used. The first follow-up took place between 2010 and 2012 and included the serum metabolites measured in 855 participants as detailed above. Six participants were excluded due to missing information or implausible dietary intake values. Thus, the final study sample comprised 849 participants. Written informed consent was obtained from all study participants, and ethics approval was given by the Ethics Committee of the Medical Faculty of the University of Kiel, Germany (approval number A156/3). All used information were taken from the PopGen Biobank (Schleswig-Holstein, Germany) and can be requested by a Material Data Access Form. Further information is available at http://www.uksh.de/p2n/Information+for+Researchers.html.

### 4.2. Assessment of Diet

At the first follow-up examination (2010–2012) dietary intake of the participants was assessed by a self-administered, semi quantitative food frequency questionnaire (FFQ) [39]. Based on the frequency and the portion size reported in the FFQ, the daily food intake in grams was calculated for each food item and each study participant. Further, alcohol consumption was calculated from the FFQ using definitions of alcoholic beverages in Germany. The resulting number of glasses consumed was converted into grams of alcohol per day. Overall, the FFQ included 141 food items and beverages for which participants reported their consumption during the previous 12 months. In total, 14 food items were excluded because they were not consumed (*n* = 2) or due to zero variance (*n* = 12). Based on comparable nutritional composition or culinary usage, we categorized the remaining food items (*n* = 127) into 41 food groups (Supplemental Table S7). 

### 4.3. Assessment of Other Lifestyle Variables and Definitions

Body weight, height, and waist circumferences were assessed by trained staff [38,40]. Waist circumferences were measured at the midpoint between lower costal margin and the superior iliac crest [40]. To determine body weight and height, the participants were weighed in light clothing and without shoes. The BMI was calculated as kg/m^2^. A validated questionnaire was used to evaluate and calculate physical activity in metabolic equivalent (MET)-h/week [40,41]. Hypertension was defined when one of the following criteria was fulfilled: abnormal blood pressure (≥140 mmHg or ≥90 mmHg), use of antihypertensive medication or self-reported hypertension. Further, diabetes type II was defined based on abnormal glucose markers (HbA1c ≥ 6.5% and glucose ≥ 126) or use of medication or self-reported diabetes.

### 4.4. Profiling of the Serum Metabolome

Fasting blood samples were obtained from participants. Samples were drawn into serum separator tubes (Sarstedt AG, Nürnbrecht, Germany), centrifuged, aliquoted, and stored (at −80 °C) prior analysis. Overall, serum metabolites were relative quantified in samples from 855 participants, at the Helmholtz Zentrum München, by a LC-MS/MS based untargeted metabolomics approach, as described elsewhere [42,43]. In brief, 100 μL of the sample were pipetted into a 2 mL 96-well plate. Further, two reference samples (human reference plasma and a pool of human serum samples) were extracted and used as technical replicates in the data to evaluate process variability. In addition, 100 μL of water served as process blanks. Serum metabolites were extracted from the serum samples with 475 µL methanol. After centrifugation, four aliquots of the supernatant, each 100 μL, were split onto two 96-well microplates. Two aliquots were used for LC-MS/MS analysis in positive and negative electrospray ionization mode and two further aliquots were retained as reserves. After drying of the samples on a TurboVap 96 (Caliper Life Sciences GmbH (formerly Zymark), Mainz, Germany) and prior to LC-MS/MS, samples in positive ion mode and samples in negative ion mode were reconstituted. Reconstitution solvents contained further internal standards that were used as retention reference markers and allowed monitoring of instrument performance. Liquid handling was carried out on a Hamilton Microlab STAR robot (Hamilton Bonaduz AG, Bonaduz, Switzerland) and samples were analyzed by a linear ion trap LTQ XL mass spectrometer (Thermo Fisher Scientific, Dreieich, Germany) coupled with a Waters Acquity UPLC system (Waters GmbH, Eschborn, Germany). Further, two separate columns were utilized either for acidic or for basic mobile phase conditions. The columns were developed over 11 min run time at 350 µL/min flow rate. Eluent flow was directed through the ESI source of the LTQ XL mass spectrometer. Mass spectrometer analysis varied between MS and data-dependent MS/MS scans by dynamic exclusion with a scan range between 80–1000 *m*/*z*. In total, 645 metabolites were detected by LC-MS/MS and identified using Metabolon^®^’s (Morrisville, NC, USA) library database based on the retention index and MS and MS/MS spectra [42]. Retention index of a compound is a number, obtained by interpolation, relating the adjusted retention time of the compound to the retention times of two standards eluted before and after the peak of the compound. Compared to the retention time of a compound, the retention index is not shifted by factors, such as the different manufacture lot of chromatographic columns. 

Supplemental Table S8 gives an overview of the relative quantified metabolites. Metabolites were categorized into one of the following metabolite classes: amino acid (and derivatives), carbohydrate, cofactors and vitamins, energy metabolites, lipids, nucleotides, peptides, xenobiotics, and unknown compounds (i.e., compounds without annotated chemical structure). Raw ion counts of metabolites were normalized by the median value of the samples run day to account for instrumental drift in the analytical measurements. We excluded metabolites with more than 80% of missing values (*n* = 81). Thus, 564 metabolites were included in the present analysis. The remaining missing values were multiple imputed by the random forest method using the R package “mice” [44] with 10 imputations and five iterations. After imputation, metabolite measurements were natural log transformed.

### 4.5. Literature Search on Food Group-Metabolite Associations

In order to confirm previously reported food group-metabolite associations, we conducted a systematic literature search using the PubMed database to identify food-metabolite associations that have been reported in at least two independent observational studies. The corresponding search term is shown in Supplemental Table S9. For the present analysis, to ensure that the extracted studies were comparable to the present study in terms of design, sample profiled, and the metabolomics approach, we considered all observational studies that investigated blood metabolites and used an untargeted metabolomics approach. 

### 4.6. Statistical Analysis

Follow-up characteristics of the participants are presented as means (with standard deviations (SD)) or as medians (with interquartile ranges [IQR]) for continuous variables and as numbers (percentages) for categorical variables. Differences between sexes were assessed by *t*-tests for continuous variables and chi-square tests for categorical variables. The absence of a batch effect for this study population was already demonstrated in a previous study [45]. Based on literature knowledge, we selected covariates that were related to both food intake and metabolites. A directed acyclic graph (DAG) was used to determine the minimum set of covariates and consisted of the following covariates: age, BMI, BMI-adjusted waist circumference, education, occupation, smoking, physical activity as (MET)-h/week, prevalent diseases (hypertension, diabetes, coronary heart diseases (CHD), stroke, and cancer), menopausal status (women), and total energy intake. 

### 4.7. Replication of Food-Metabolite Associations

We fitted a linear regression model for each food group-metabolite association obtained from the systematic literature search. In the analysis of food groups that were associated with more than one metabolite, we adjusted *p*-values for multiple testing using the Bonferroni correction. All models were adjusted for the minimum adjustment set of covariates as aforementioned. Further, we integrated an interaction between food group and sex to adjust for possible differences in food-metabolite associations between sexes. For a simplified interpretation on the original scale of the metabolites, the effect estimates were back transformed.

### 4.8. Identification of Novel Food-Metabolite Associations

To identify potential new food group related metabolites, we conducted sex-specific analyses. We randomly split the data of both men and women into training and test datasets, where both training datasets (male and female) included 50% of the observations and both test datasets included the remaining 50% of the observations. We used linear regression models to assess the associations between all food groups (*n* = 41) available in our study and all available single serum metabolites (*n* = 564) in the training dataset. Food-metabolite associations with a nominal *p*-value ≤ 0.05 were used to generate hypotheses which were further tested in the test dataset. We adjusted the *p*-values obtained from the models on the test dataset for multiple testing using the Bonferroni correction. All models were adjusted for the minimum adjustment set of covariates as aforementioned. Again, for a simplified interpretation on the original scale, the effect estimates were back transformed.

We performed all analyses with the open source software R (version 3.5.3) [46].

## 5. Conclusions

In total, we were able to confirm 26 associations of specific metabolites with alcohol, butter, coffee, fish, chocolate, poultry, and wine previously reported in the literature. Overall, the observed findings suggests that these metabolites could be robust biomarkers for their associated food intakes. Moreover, we found sex-specific associations of coffee and fish with four metabolites. This novel finding suggests that sex-specificity should be considered in dietary biomarker research.

## Figures and Tables

**Table 1:** Characteristics of the study population at the time of follow-up (2010–2012) in PopGen by sex ^1^.

Participant Characteristics	Total (*n* = 849)	Men (*n* = 485)	Women (*n* = 364)	*p*-Value
Sociodemographic and lifestyle				
Age ^2^, yrs.	62.2 [16.5]	62.0 [15.3]	62.3 [18.2]	0.61
BMI ^2^, kg/m^2^	26.8 [5.4]	27.1 [4.5]	26.2 [6.8]	0.02
Waist circumference ^2^, cm	96.0 [16.9]	100.0 [14.5]	89.7 [19.0]	<0.01
High education ^4^	277 (32.6%)	186 (38.4%)	91 (25%)	<0.01
Full time employment ^4^	263 (31%)	199 (41%)	64 (17.6%)	<0.01
Current smokers ^4^	108 (12.7%)	63 (13%)	45 (12.4%)	<0.01
Physical activity, MET-h/week ^1,3^	100.9 (61.5)	96.4 (64.0)	107.1 (57.6)	0.01
Prevalent diseases ^4^				
Hypertension	638 (75.1%)	386 (79.6%)	252 (69.2%)	<0.01
CHD	72 (8.5%)	58 (12%)	14 (3.8%)	<0.01
Stroke	16 (1.9%)	9 (1.9%)	7 (1.9%)	1
Diabetes	101 (11.9%)	72 (14.8%)	29 (8%)	<0.01
Cancer	137 (16.1%)	84 (17.3%)	53 (14.6%)	0.29
Dietary intake ^2^, g/d				
Alcohol	8.9 [15.3]	12.5 [18.1]	5.3 [10.0]	<0.01
Butter	7.9 [12.7]	9.0 [16.7]	7.0 [9.9]	<0.01
Chocolate	9.0 [10.9]	11.0 [12.0]	9.0 [9.2]	0.01
Coffee	370.8 [477.3]	390.3 [490.3]	356.8 [121.4]	<0.01
Fish	21.5 [29.5]	31.4 [27.9]	21.5 [23.4]	<0.01
Mushrooms	3.7 [0.9]	3.8 [0.9]	3.6 [0.8]	<0.01
Liquor	0.0 [1.3]	0.0 [1.3]	0.0 [1.3]	0.21
Poultry	11.5 [14.0]	11.7 [13.4]	11.3 [15.3]	0.96
Red meat	51.5 [44.7]	68.0 [48.9]	29.4 [25.3]	<0.01
Wine	32.1 [86.6]	37.0 [92.0]	30.5 [85.0]	0.12
Total energy intake ^2^, kJ/day	8915.7 [3545.3]	10,059.9 [3616.2]	7751.9 [2332.4]	<0.01

^1^ Mean and SD in parenthesis; ^2^ Median and the IQR in square bracket; ^3^ Metabolic equivalent (MET) in hours per week; ^4^ number and percentages. Abbreviations: BMI, body mass index; CHD, Coronary heart diseases; cm, centimeter; kg, kilogram; m, meter; yrs., years; Available *n* values differ due to missing data (education, *n* = 3; employment, *n* = 3; smoking status, *n* = 23; CHD, *n* = 12; Apoplexy, *n* = 4; diabetes, *n* = 35, cancer, *n* = 5).

**Table 2:** Food groups associated with metabolites in at least two independent observational studies (table includes search results up to 16 December 2019).

Food Groups	Metabolites	Observational Study
Apples and pears ^a^	Threitol ^b^	[6,15]
Fruit juice ^a^	Stachydrine or proline betaine	[6,9]
Juices ^a^	4-Hydroxyproline betaine/betonicine ^b^	[8,15]
Mushrooms	Ergothioneine	[6,16]
Fish	X-02269	[8,16]
	CMPF	[7,8,15,16]
	DHA	[7,8,15,16]
	EPA	[7,16]
	1-Docosahexaenoyl-GPC (22:6) *	[7,16]
Fish and seafood ^a^	DHA	[9,17]
	CMPF	[9,17]
	EPA	[9,17]
Shellfish ^a^	CMPF	[7,8,16,17]
	X-02269	[8,16]
Nuts ^f^	Tryptophan betaine	[8,16]
	4-Vinylphenol sulfate	[8,16]
Peanuts ^a^	Tryptophan betaine	[7,16]
	4-Vinylphenol sulfate	[7,16]
Milk	Galactonate ^b^	[15,16]
	Phenylacetylglycine ^b^	[15,16]
Butter	Caprate 10:0	[15,16]
	15-Methylpalmitate isobar with 2-methylpalmitate ^b^	[6,7]
	10-Undecenoate (11:1n1)	[6,7,16]
	Pentadecanoate 15:0 ^b^	[6,7]
Chocolate	7-Methylxanthine	[6,16]
	Theobromine	[6,7,16]
Alcohol ^e^	Ethyl glucuronide ^c^	[7,8,16]
	5-α-Androstan-3β,17β-diol disulfate	[7,15,16]
	4-Androsten-3β,17β-diol disulfate 1 *	[7,15]
	α-Hydroxyisovalerate	[15,16]
	Ergothioneine	[15,16]
	CMPF	[15,16]
	2,3-Dihydroxyisovalerate ^b^	[15,16]
	5α-Androstan-3α,17β-diol disulfate ^b^	[15,16]
Liquor	Ethyl glucuronide ^c^	[7,8,15,16]
	5α-Androstan-3α,17β-diol disulfate ^b^	[15,16]
	α-Hydroxyisovalerate	[15,16]
	5α-Androstan-3β,17β-diol disulfate	[15,16]
Coffee ^d^	1,3,7-Trimethylurate	[8,9,16,18]
	1,3-Dimethylurate ^b^	[8,16]
	1,7-Dimethylurate	[8,9,16,18]
	1-Methylurate	[9,16,18]
	1-Methylxanthine	[6,7,8,9,16,18]
	3-(3-Hydroxyphenyl)-propionate	[16,18]
	3-Hydroxyhippurate	[16,18]
	4-Vinylguaiacolsulfate ^b^	[16,18]
	3-Hydroxypyridine sulfate ^b^	[6,16]
	AAMU	[9,16,19]
	3-Methyl catechol sulfate (1)	[6,16]
	Caffeine	[8,9,16,18,19]
	Catechol sulfate	[6,7,8,16,18,19]
	Cinnamoylglycine	[16,18]
	Dihydroferulic acid ^b^	[15,16]
	N-(2-Furoyl)-glycine	[7,8,16,18]
	O-Methylcatechol sulfate ^b^	[6,16]
	Paraxanthine	[7,8,9,16,18,19]
	Quinate ^b^	[6,7,8,9,16,18,19]
	Theophylline	[8,16,18,19]
	Trigonelline N ′-methylnicotinate ^b^	[7,8,16,18,19]
	Cyclo(leu-pro)	[18,19]
	Hippurate	[16,19]
	X-12230 ^b^	[6,16,18]
	X-12329 ^b^	[8,16,18]
	X-12816	[6,8,16,18]
	X-14473	[6,8,16,18]
	X-17185	[8,16,18]
Decaffeinated coffee ^a^	1,7-Dimethylurate	[8,16]
Tea	Theanine ^b^	[15,16]
Poultry	Pyroglutamine	[6,7]
	3-Methylhistidine	[15,16]
Soymilk ^a^	4-Ethylphenylsulfate	[6,16]
Meat ^f^	Pyroglutamine	[6,17]
	Trans-4-hydroxyproline	[6,17]
Red meat	Pyroglutamine	[6,15]
	X-11381	[6,16]
Wine	Scyllo-inositol ^b^	[6,7]
	X-01911	[6,16]
	X-11795	[6,16]
	Piperine	[6,16]
	Ethyl glucuronide ^b^	[15,16]
	CMPF	[15,16]
	2,3-Dihydroxyisovalerate ^b^	[15,16]

Not assessed in our study due to missing information about: ^a^ Food group; ^b^ missing metabolite; ^c^ excluded due to more than 80% missings; ^d^ including caffeinated coffee; ^e^ total alcohol intake calculated from the FFQ; ^f^ not assessed due to highly heterogeneous definition in the studies. * No pure compound was available, but we are confident in its identity. Abbreviations: AAMU, 5-acetylamino-6-amino-3-methyluracil; CMPF, 3-carboxy-4-methyl-5-propyl-2-furanpropionate; DHA, docosahexaenoic acid; EPA, eicosapentaenoic acid; GPC, glycerophosphocholine.

**Table 3:** Confirmed food-metabolite associations for the food groups butter, poultry, and wine.

Food Group	Metabolite	Subpathway	Estimate (95% CI) in %	*p*-Value *
Butter	undecenoate (11:1n1)	Medium chain fatty acid	0.31 (0.06, 0.55)	0.03
Poultry	3-methylhistidine	Histidine metabolism	1.08 (0.23, 1.93)	0.03
Wine	X-11795	unknown	0.04 (0.02, 0.06)	<0.01

Estimates were generated from linear regression models with natural log-transformed metabolites as the dependent variables, and butter or poultry or wine as the independent variable. * Adjusted for multiple testing by Bonferroni correction.

**Table 4:** Food-metabolite associations in the identification analysis.

Food Group	Metabolites	Subpathway	Estimate (95% CI) in %	*p*-Value *
Coffee	paraxanthine ^1^	Xanthine metabolism	0.08 (0.04, 0.11)	0.03
	X-144732 ^2^	Unknown	0.13 (0.07, 0.19)	0.05
Fish	EPA ^1^	Polyunsaturated fatty acid	0.68 (0.39, 0.98)	0.40
	X-02269 ^1^	Unknown	1.12 (0.67, 1.58)	0.01

Estimates were generated from linear regression models with natural log-transformed metabolites as the dependent variables, and butter or poultry or wine as the independent variable. ^1^ Identified in men; ^2^ identified in women. * Adjusted for multiple testing by Bonferroni correction.

## Supplementary Materials

The following are available online at https://www.mdpi.com/2218-1989/10/11/468/s1; Table S1: Self-reported usual dietary intake in g/d of the study sample, Table S2: Characteristics of the included studies, Table S3: Replicated food-metabolite associations and their corresponding back transformed estimator and confidence interval, Table S4: Food-metabolite associations in the identification analysis for women and their corresponding back transformed estimator and confidence interval found in the train dataset, Table S5: Food-metabolite associations in the identification analysis for men and their corresponding back transformed estimator and confidence interval found in the train dataset, Table S6: Food-metabolite associations in the identification analysis and their corresponding back transformed estimator and confidence interval found in the test dataset, Table S7: Food groups used in the present analysis, Table S8: Annotation and metabolite descriptions, Table S9: Applied search term.
