# Supplementary material for: Blood Metabolomic Profiling Confirms and Identifies Biomarkers of Food Intake
Source: Metabolites. 2020 Nov 17;10(11):468. doi: 10.3390/metabo10110468 (PMC7698441; doi:10.3390/metabo10110468)
Supplement: Supplementary file 1 [file metabolites-10-00468-s001.zip › metabolites-977557-supplementary-proof/Supplement_Metabolites_revision.pdf]

Supplementary data

Table S1: Self-reported usual dietary intake in g/d of the study sample

Food group	Total	Men	Women
	n = 849	n = 485	n = 364
Beer Cider	38.31 [138.4]	71.25 [201.45]	8.2 [16.4]
Bread	89.89 [44.89]	100.97 [44.25]	75.78 [34.47]
Butter	7.87 [12.71]	9.05 [16.67]	6.96 [9.9]
Cabbages	22.9 [13.22]	25.07 [13.71]	20.69 [11.82]
Cake cookies	51.6 [50.58]	52.66 [50.74]	48.2 [47.93]
Cheese	33.12 [21.19]	33.28 [20.99]	32.77 [20.33]
Chocolate	8.97 [10.86]	11 [11.95]	8.97 [9.23]
Coffee	370.78 [478.18]	390.28 [490.3]	356.83 [121.36]
Dairy products	94.04 [86.57]	89.14 [84.69]	100.74 [89.08]
Eggs	18.34 [11.97]	18.14 [11.4]	19.92 [12.28]
Fish	21.45 [29.52]	31.43 [27.88]	21.45 [23.41]
Fruit and vegetable juice	59.35 [141.45]	70.29 [180.45]	43.16 [100.85]
Fruiting vegetables	80.41 [42.17]	80.66 [45.75]	80.27 [39.93]
Fruits	180.4 [163.23]	165.06 [124.23]	200.54 [196.89]
Leafy vegetables	12.49 [13.49]	13.08 [15.25]	12.1 [11.53]
Legumes	1.82 [2.49]	1.82 [4.98]	1.2 [0]
Liqour[1]	0 [1.3]	0 [1.3]	0 [1.3]
Margarines	10.95 [15.97]	14.19 [17.45]	7.3 [10.49]
Milk	53.55 [172.16]	55.07 [193.93]	52.49 [121.08]
Miscellaneous	4.57 [2.5]	4.61 [2.4]	4.48 [2.68]
Mushrooms[2]	3.74 [0.91]	3.81 [0.89]	3.6 [0.83]
Nuts and seeds	2.15 [3.99]	2.87 [2.49]	2.15 [4.16]
Offals	0.92 [2.25]	1.17 [2.25]	0.13 [2.18]
Other alcoholic beverages	3.35 [9.77]	4.01 [12.62]	3.28 [6.58]
Other cereals	7.3 [8.1]	8.07 [10.58]	6.19 [6.12]
Other fats	1.78 [1.11]	2.01 [1.23]	1.53 [0.78]
Other fruits	2.79 [2.82]	2.52 [2.13]	3.58 [4.02]
Other non-alcoholic drinks	814.27 [1014.32]	807.04 [959.47]	1095.6 [796.46]
Other vegetables	36.2 [16.75]	36.63 [16.29]	35.88 [16.84]
Pasta rice	36.5 [30.83]	36.48 [22.49]	47.13 [28.94]
Potatoes	84.2 [69.18]	94.44 [70.11]	68.75 [64.21]
Poultry	11.62 [14.08]	11.72 [13.66]	11.25 [15.34]
Processed meat	43.16 [37.66]	55.77 [35.33]	27.87 [23.01]
Red meat	38.36 [49.43]	56.77 [61.04]	25.11 [28.49]
Root vegetables	18.3 [14.5]	18.17 [14.21]	18.94 [15.52]
Sauces	47.68 [29.85]	49.55 [29.6]	46.24 [29.93]

Supplementary data

Food group	Total	Men	Women
Soft drinks	13.63 [43.89]	14.29 [152.14]	13.63 [8.1]
Soups	32.96 [26.57]	34.48 [22.43]	30.44 [30.6]
Spirits	0.26 [0.77]	0.26 [2.22]	0 [0.26]
Sugar	35.06 [32.27]	36.6 [37.22]	32.21 [32.12]
Tea	108.73 [275.03]	58.33 [263.35]	254.27 [362.71]
Vegetable oil	7.74 [5.41]	7.91 [5.7]	7.51 [5.02]
Wine	32.48 [87.84]	37.83 [92.64]	30.46 [84.95]

Food items of the food groups [1]other alcoholic beverages and [2]other vegetables; Median and the interquartile range in square bracket

Supplementary data

Table S2: Characteristics of the included studies

Study Number	Author, year, country*, [reference]	Parent study	Number of participants (sex)	Dietary assessment method	Bio sample	Number of measured metabolites
1	Playdon et al., 2017, FI [15]	PLCO Cancer Screening Trial	1.242 (only women)	FFQ	Serum non-fasting samples	617 (only known metabolites)
2	Pallister et al., 2016, UK [6]	TwinsUK registry	3.559 (only women)	FFQ	Fasting serum and plasma samples	601 (442 known and 159 unknown)
3	Zheng et al., 2014, US [9]	ARIC Study	1.977 (702 men, 1275 women)	FFQ	Fasting serum samples	356 (only known metabolites included)
4	Playdon et al., 2016, US [8]	Colon adenoma case–control study	253 (77% men with non-fasting serum and 100% with non-fasting urine samples)	FFQ	Non-fasting serum samples and 12-h overnight non-fasting urine samples	676 serum metabolites and 848 urine metabolites
5	Wang et al., 2018, NO [16]	CPS-II Nutrition Cohort	1.369 (only women)	FFQ	Non-fasting serum samples	1186 (no information on known/unknown metabolites)
6	Guertin et al., 2014, US [7]	PLCO Cancer Screening Trial	502 (281 men and 221 women)	FFQ	Serum samples (no information about fasting/non-fasting available)	643 (412 known and 231 unknown metabolites)
7	Playdon et al. 2017, FI [17]	ATBC Study	1336 (only men)	FFQ	Fasting serum samples	1316 (1overall 994–1220 metabolites of which 626–722 were known)
8	Guertin et al., 2015, US [18]	PLCO Cancer Screening Trial	498 (279 men, 219 women)	FFQ	Non-fasting serum samples	657 (428 known and 229 unknown metabolites)
9	Rothwell et al. 2019, FR, DE, GR, and IT [19]	EPIC Study	451 (192 men, 259 women)	24 h dietary recall	Serum sample (no information about fasting/non-fasting available)	No information available

* DISO Country Codes (two-letter abbreviation); [1]Data from different nested case-control studies were used; Abbreviations: ARIC, Atherosclerosis Risk in Communities Study; ATBC, Alpha Tocopherol, Beta-Carotene Cancer Prevention Study cohort; EPIC, European Prospective Investigation on Cancer and Nutrition FFQ, Food Frequency Questionnaire; PLCO, Prostate, Lung, Colorectal and Ovarian Cancer Screening Trial

Supplementary data

Table S3: Replicated food-metabolite associations and their corresponding back transformed estimator and confidence interval

Food group	Metabolite	Subpathway	β-Estimator	Confidence interval (CI)	p.value
Alcohol	ergothioneine	Food Component/Plant	1.0050	(1.0009, 1.0090)	0.0170
	α-hydroxyisovalerate	Leucine, Isoleucine and Valine Metabolism	1.0058	(1.0023, 1.0092)	0.0062
	5α-androstan-3ß,17ß-diol disulfate	Steroid	1.0048	(1.0015, 1.0081)	0.0183
	4-androsten-3ß,17ß-diol disulfate 1(1)*	Steroid	1.0092	(1.0066, 1.0118)	<.0001
Butter	10-undecenoate	Medium Chain Fatty Acid	1.0031	(1.0006, 1.0055)	0.0286
Chocolate	theobromine	Xanthine Metabolism	1.0199	(1.0110, 1.0289)	<.0001
	7-methylxanthine	Xanthine Metabolism	1.0212	(1.0117, 1.0309)	<.0001
Coffee	hippurate	Benzoate Metabolism	1.0003	(1.0002, 1.0005)	1e-04
	catechol sulfate	Benzoate Metabolism	1.0003	(1.0002, 1.0004)	<.0001
	3-methyl catechol sulfate(1)	Benzoate Metabolism	1.0004	(1.0002, 1.0006)	0.0009
	X-12816	unknown	1.0007	(1.0005, 1.0010)	<.0001
	X-14473	unknown	1.0006	(1.0004, 1.0007)	<.0001
	paraxanthine	Xanthine Metabolism	1.0007	(1.0006, 1.0009)	<.0001
	theophylline	Xanthine Metabolism	1.0007	(1.0005, 1.0009)	<.0001
	1-methylxanthine	Xanthine Metabolism	1.0005	(1.0002, 1.0007)	0.0096
	1-methylurate	Xanthine Metabolism	1.0006	(1.0004, 1.0008)	<.0001
	1,7-dimethylurate	Xanthine Metabolism	1.0006	(1.0004, 1.0009)	<.0001
	5-acetylamino-6-amino-3-methyluracil (AAMU)	Xanthine Metabolism	1.0005	(1.0003, 1.0007)	0.0019
	caffeine	Xanthine Metabolism	1.0005	(1.0003, 1.0007)	<.0001
Fish	3-carboxy-4-methyl-5-propyl-2-furanpropionate (CMPF)	Fatty Acid, Dicarboxylate	1.0130	(1.0097, 1.0163)	<.0001
	1-docosahexaenoyl GPC(22:6)*	Lysolipid	1.0091	(1.0055, 1.0126)	<.0001
	eicosapentaenoic acid (EPA)	Polyunsaturated Fatty Acid (n3 and n6)	1.0063	(1.0047, 1.0079)	<.0001
	docosahexaenoic acid (DHA)	Polyunsaturated Fatty Acid (n3 and n6)	1.0065	(1.0050, 1.0080)	<.0001
	X-02269	unknown	1.0111	(1.0082, 1.0139)	<.0001
Poultry	3-methylhistidine	Histidine Metabolism	1.0108	(1.0024, 1.0193)	0.0251
Wine	X-11795	unknown	1.0004	(1.0002, 1.0006)	0.0015

Table S4: Food-metabolite associations in the identification analysis for women and their corresponding back transformed estimator and confidence interval found in the train dataset

Food group	Metabolite	Subpathway	β-Estimator	Confidence interval (CI)	p.value
Beer Cider	cyclo(leu pro)	Dipeptide	1.0004	(1.0001,1.0007)	0.0142
	cysteine-glutathione disulfide	Glutathione Metabolism	0.9996	(0.9992,0.9999)	0.0271
	palmitoleate (16:1n7)	Long Chain Fatty Acid	1.0002	(1,1.0005)	0.0363
	eicosenoate (20:1)	Long Chain Fatty Acid	1.0002	(1,1.0004)	0.0295

Supplementary data

Food group	Metabolite	Subpathway	β-Estimator	Confidence interval (CI)	p.value
	1-margaroyl-GPC (17:0)	Lysolipid	0.9995	(0.9991,0.9999)	0.03
	dihomo-linoleate (20:2n6)	Polyunsaturated Fatty Acid (n3 and n6)	1.0002	(1.0001,1.0004)	0.013
	eicosapentaenoate (EPA 20:5n3)	Polyunsaturated Fatty Acid (n3 and n6)	1.0002	(1,1.0004)	0.0408
	docosahexaenoate (DHA 22:6n3)	Polyunsaturated Fatty Acid (n3 and n6)	1.0001	(1,1.0003)	0.0428
	docosapentaenoate (n3DPA 22:5n3)	Polyunsaturated Fatty Acid (n3 and n6)	1.0002	(1,1.0004)	0.0241
	Adrenate (22:4n6)	Polyunsaturated Fatty Acid (n3 and n6)	1.0002	(1,1.0004)	0.0269
	urate	Purine Metabolism, (Hypo)Xanthine/Inosine containing	1.0000	(1,1.0001)	0.0441
	5α-androstan-3β,17β-diol disulfate	Steroid	1.0003	(1,1.0005)	0.0425
	4-androsten-3β,17β-diol disulfate(1)*	Steroid	1.0005	(1.0001,1.0008)	0.0207
	X-11315	unknown	0.9998	(0.9997,1)	0.0274
	X-11444	unknown	0.9997	(0.9994,1)	0.0491
	X-11799	unknown	1.0003	(1.0001,1.0006)	0.0157
	X-14473	unknown	1.0004	(1.0001,1.0008)	0.0106
	X-21365	unknown	0.9999	(0.9998,1)	0.0374
	theobromine	Xanthine Metabolism	0.9995	(0.9992,0.9999)	0.0058
Butter	17-methylstearate	Fatty Acid, Branched	1.0107	(1.0027,1.0187)	0.0098
	15-methylpalmitate	Fatty Acid, Branched	1.0135	(1.0053,1.0217)	0.0017
	margarate (17:0)	Long Chain Fatty Acid	1.0031	(1.0001,1.0062)	0.0496
	10-nonadecenoate (19:1n9)	Long Chain Fatty Acid	1.0044	(1.0006,1.0083)	0.0246
	X-11261	unknown	0.9926	(0.9872,0.9981)	0.0123
	X-11438	unknown	1.0107	(1.0017,1.0198)	0.0226
Cake cookies	4-androsten-3β,17β-diol disulfate(1)*	Steroid	0.9976	(0.9957,0.9995)	0.0155
	X-11372	unknown	1.0016	(1.0004,1.0028)	0.0092
Cheese	catechol sulfate	Benzoate Metabolism	1.0040	(1.0006,1.0074)	0.0236
	4-methylcatechol sulfate	Benzoate Metabolism	1.0064	(1.0003,1.0125)	0.0423
Chocolate	2-aminobutyrate	Methionine, Cysteine, SAM and Taurine Metabolism	0.9965	(0.9932,0.9998)	0.0424
Chocolate	theobromine	Xanthine Metabolism	1.0227	(1.0067,1.039)	0.0098
	3-methylxanthine	Xanthine Metabolism	1.0160	(1.0007,1.0316)	0.0461
	7-methylxanthine	Xanthine Metabolism	1.0206	(1.0036,1.0379)	0.0251
Coffee	catechol sulfate	Benzoate Metabolism	1.0002	(1.0001,1.0004)	0.0142
	4-vinylphenol sulfate	Benzoate Metabolism	1.0004	(1.0001,1.0007)	0.0194
	3-methylcatechol sulfate(1)	Benzoate Metabolism	1.0004	(1.0001,1.0007)	0.0167
	cyclo(leu pro)	Dipeptide	1.0003	(1,1.0006)	0.0314
	N-(2-furoyl)glycine	Food Component/Plant	1.0003	(1,1.0006)	0.0466

Supplementary data

Food group	Metabolite	Subpathway	β-Estimator	Confidence interval (CI)	p.value
	X-12816	unknown	1.0008	(1.0005,1.0011)	<.0001
	X-14465	unknown	1.0004	(1.0001,1.0007)	0.0219
	X-14473	unknown	1.0007	(1.0004,1.001)	<.0001
	X-16125	unknown	1.0006	(1.0003,1.0009)	0.0011
	dimethylarginine(SDMA+ADMA)	Urea cycle; Arginine and Proline Metabolism	0.9997	(0.9994,1)	0.0373
	paraxanthine	Xanthine Metabolism	1.0008	(1.0005,1.0011)	<.0001
	theophylline	Xanthine Metabolism	1.0008	(1.0005,1.001)	<.0001
	1-methylurate	Xanthine Metabolism	1.0006	(1.0003,1.0008)	3e-04
	1,7-dimethylurate	Xanthine Metabolism	1.0006	(1.0002,1.001)	0.0036
	5-acetylamino-6-formylamino-3 methyluracil	Xanthine Metabolism	1.0004	(1,1.0007)	0.0385
	5-acetylamino-6-amino-3 methyluracil (AAMU)	Xanthine Metabolism	1.0005	(1.0002,1.0008)	0.0049
	caffeine	Xanthine Metabolism	1.0006	(1.0003,1.0009)	2e-04
Dairy products	X-21365	unknown	1.0007	(1.0002,1.0012)	0.0054
Eggs	indoleacetate	Tryptophan Metabolism	1.0045	(1.0001,1.0089)	0.0455
	X-18554	unknown	1.0090	(1.0009,1.0171)	0.0294
Fish	creatine	Creatine Metabolism	1.0026	(1.0003,1.0049)	0.0337
	3-carboxy-4-methyl-5-propyl-2-furanpropanoate(CMPF)	Fatty Acid, Dicarboxylate	1.0147	(1.0094,1.0199)	<.0001
	pyroglutamine*	Glutamate Metabolism	0.9966	(0.9941,0.9992)	0.0133
	erucate (22:1n9)	Long Chain Fatty Acid	1.0059	(1.0007,1.0111)	0.0295
	eicosenoate (20:1)	Long Chain Fatty Acid	1.0034	(1.0007,1.0062)	0.0184
	1-docosahexaenoyl-GPC(22:6)*	Lysolipid	1.0089	(1.0042,1.0135)	2e-04
	1-eicosapentaenoyl-GPC(20:5)*	Lysolipid	1.0052	(1.0018,1.0086)	0.0041
	1-docosahexaenoyl-GPE(22:6)*	Lysolipid	1.0033	(1.001,1.0056)	0.0078
	2-aminobutyrate	Methionine, Cysteine, SAM and Taurine Metabolism	1.0019	(1.0003,1.0035)	0.0302
	eicosapentaenoate (EPA 20:5n3)	Polyunsaturated Fatty Acid (n3 and n6)	1.0055	(1.0028,1.0081)	2e-04
	docosahexaenoate (DHA 22:6n3)	Polyunsaturated Fatty Acid (n3 and n6)	1.0064	(1.004,1.0087)	<.0001
	docosapentaenoate (n3DPA 22:5n3)	Polyunsaturated Fatty Acid (n3 and n6)	1.0028	(1.0004,1.0052)	0.0254
	linolenate [alpha or gamma; (18:3n3 or 6)]	Polyunsaturated Fatty Acid (n3 and n6)	1.0026	(1.0001,1.005)	0.041
	X-02269	unknown	1.0127	(1.0087,1.0167)	<.0001
Fruiting vegetables	stearate (18:0)	Long Chain Fatty Acid	0.9995	(0.999,1)	0.045
	androsterone sulfate	Steroid	0.9986	(0.9973,1)	0.0483
Fruits	oxalate (ethanedioate)	Ascorbate and Aldarate Metabolism	1.0003	(1.0001,1.0006)	0.006
	stachydrine	Food Component/Plant	1.0007	(1.0001,1.0013)	0.0208

Supplementary data

Food group	Metabolite	Subpathway	β-Estimator	Confidence interval (CI)	p.value
	methyl-beta-glucopyranoside	Fructose, Mannose and Galactose Metabolism	1.0010	(1.0001,1.0018)	0.0332
	X-11315	unknown	1.0004	(1.0002,1.0007)	0.0014
	X-17145	unknown	1.0010	(1.0002,1.0019)	0.0265
Leafy vegetables	catechol sulfate	Benzoate Metabolism	1.0049	(1.0008,1.009)	0.0214
	Palmitoleate (16:1n7)	Long Chain Fatty Acid	0.9967	(0.9935,1)	0.0487
	X-16132	unknown	0.9902	(0.9831,0.9974)	0.0094
	X-17259	unknown	1.0069	(1.0001,1.0138)	0.0492
Margarines	X-11261	unknown	1.0086	(1.0044,1.0127)	1e-04
	X-11478	unknown	1.0125	(1.0043,1.0208)	0.0035
	X-11521	unknown	1.0098	(1.0025,1.0172)	0.0097
Milk	2-aminoheptanoate	Fatty Acid, Amino	0.9988	(0.9978,0.9998)	0.0328
	acisoga	Polyamine Metabolism	0.9997	(0.9994,1)	0.037
	X-21365	unknown	1.0004	(1.0002,1.0006)	0.0012
Miscellaneous	Catechol sulfate	Benzoate Metabolism	1.0208	(1.0022,1.0396)	0.0319
Nuts and seeds	4-hydroxyhippurate	Benzoate Metabolism	1.0208	(1.0013,1.0407)	0.0385
	X-11315	unknown	1.0084	(1.0006,1.0162)	0.0377
Offals	isovalerate	Leucine, Isoleucine and Valine Metabolism	0.9693	(0.9462,0.9931)	0.0125
Other vegetables	bilirubin (Z,Z)	Hemoglobin and Porphyrin Metabolism	0.9932	(0.987,0.9994)	0.0365
Other vegetables	bilirubin (E,E)*	Hemoglobin and Porphyrin Metabolism	0.9965	(0.9933,0.9998)	0.0418
Potatoes	X-16136	unknown	1.0032	(1.0001,1.0062)	0.0455
	X-19429	unknown	1.0037	(1.0007,1.0067)	0.019
Processed meat	pyroglutamine*	Glutamate Metabolism	0.9971	(0.9946,0.9996)	0.034
Sauces	3-(4-hydroxyphenyl)lactate	Phenylalanine and Tyrosine Metabolism	0.9950	(0.9906,0.9995)	0.0324
Spirits	4-androsten-3β,17β-diol disulfate(1)*	Steroid	1.0220	(1.0042,1.0402)	0.0183
Sugar	creatine	Creatine Metabolism	0.9977	(0.9955,0.9998)	0.0382
	stearoylcarnitine	Fatty Acid Metabolism(Acyl Carnitine)	1.0058	(1.0004,1.0112)	0.043
	pyroglutamine*	Glutamate Metabolism	1.0032	(1.0005,1.0059)	0.0266
	1-dihomo-linolenoyl-GPC(20:3n3or6)*	Lysolipid	1.0022	(1.0001,1.0042)	0.0427
	1-palmitoyl-GPC (16:0)	Lysolipid	1.0012	(1.0001,1.0024)	0.0391
	1-linoleoyl-GPC (18:2)	Lysolipid	1.0020	(1.0002,1.0039)	0.039
	2-palmitoyl-GPC (16:0)*	Lysolipid	1.0024	(1.0004,1.0043)	0.019
	2-stearoyl-GPC (18:0)*	Lysolipid	1.0022	(1.0004,1.0041)	0.02
	1-pentadecanoyl-GPC (15:0)*	Lysolipid	1.0079	(1.0021,1.0138)	0.0113
Tea	indolepropionate	Tryptophan Metabolism	1.0003	(1,1.0006)	0.0317
	X-09789	unknown	1.0004	(1.0001,1.0008)	0.0148
	X-14473	unknown	0.9995	(0.9991,0.9998)	0.0077

Supplementary data

Food group	Metabolite	Subpathway	β-Estimator	Confidence interval (CI)	p.value
	X-18901	unknown	1.0003	(1,1.0006)	0.0305
	N-delta-acetylornithine*	Urea cycle; Arginine and Proline Metabolism	1.0003	(1,1.0007)	0.0396
Vegetable oil	catechol sulfate	Benzoate Metabolism	1.0111	(1.0013,1.0209)	0.0276
Wine	eicosapentaenoate (EPA 20:5n3)	Polyunsaturated Fatty Acid (n3 and n6)	1.0006	(1.0001,1.0011)	0.0432
	X-11372	unknown	0.9996	(0.9992,0.9999)	0.0266
	X-11787	unknown	0.9998	(0.9997,1)	0.047
	X-11880	unknown	0.9995	(0.9992,0.9999)	0.0244

[1] Other cereals includes: Flour flakes starch, breakfast cereals, dough; Abbreviations: CI, confidence interval

Supplementary data

Table S5: Food-metabolite associations in the identification analysis for men and their corresponding back transformed estimator and confidence interval found in the train dataset

Food group	Metabolite	Subpathway	β-Estimator	Confidence interval (CI)	p.value
Beer Cidre	isobutyrylcarnitine	Leucine, Isoleucine and Valine Metabolism	0.9998	(0.9996,1)	0.0188
	eicosapentaenoate (EPA 20:5n3)	Polyunsaturated Fatty Acid (n3 and n6)	1.0002	(1,1.0003)	0.0462
	docosapentaenoate (n3DPA 22:5n3)	Polyunsaturated Fatty Acid (n3 and n6)	1.0002	(1,1.0004)	0.0291
	4-androsten-3β,17β diol disulfate(1)*	Steroid	1.0004	(1.0001,1.0008)	0.0187
	X-11799	unknown	1.0004	(1.0001,1.0007)	0.0197
	X-14473	unknown	1.0003	(1.0001,1.0006)	0.0245
	theobromine	Xanthine Metabolism	0.9996	(0.9992,0.9999)	0.0214
Butter	15-methylpalmitate	Fatty Acid, Branched	1.0121	(1.001,1.0234)	0.0373
	X-11261	unknown	0.9913	(0.9849,0.9976)	0.0098
Cake cookies	pipecolate	Lysine Metabolism	0.9985	(0.997,0.9999)	0.0403
	4-androsten-3β,17β-diol disulfate(1)*	Steroid	0.9976	(0.9952,1)	0.0496
	X-11372	unknown	1.0018	(1.0003,1.0032)	0.0187
Cheese	2 methylbutyrylcarnitine (C5)	Leucine, Isoleucine and Valine Metabolism	1.0029	(1.0001,1.0058)	0.0464
	X-12544	unknown	1.0094	(1.0011,1.0178)	0.0292
Chocolate	7-methylxanthine	Xanthine Metabolism	1.0259	(1.0043,1.048)	0.0257
Coffee	hippurate	Benzoate Metabolism	1.0004	(1,1.0008)	0.0499
	catecholsulfate	Benzoate Metabolism	1.0003	(1.0001,1.0005)	0.009
	4-vinylphenol sulfate	Benzoate Metabolism	1.0004	(1.0001,1.0008)	0.0238
	3-methylcatechol sulfate (1)	Benzoate Metabolism	1.0005	(1.0001,1.0008)	0.0071
	phenylcarnitine*	Chemical	1.0004	(1,1.0008)	0.0493
	X-12816	unknown	1.0008	(1.0004,1.0012)	0.0017
	X-14473	unknown	1.0005	(1.0002,1.0008)	0.0023
	X-16125	unknown	1.0007	(1.0003,1.0011)	0.0011
	paraxanthine	Xanthine Metabolism	1.0007	(1.0004,1.0011)	1e-04
	theophylline	Xanthine Metabolism	1.0008	(1.0004,1.0011)	3e-04
	1-methylurate	Xanthine Metabolism	1.0006	(1.0002,1.001)	0.0037
	caffeine	Xanthine Metabolism	1.0005	(1.0001,1.0009)	0.0138
Dairy products	stachydrine	Food Component/Plant	1.0023	(1.0006,1.0041)	0.0121
	X-21365	unknown	1.0008	(1.0001,1.0015)	0.0225
Eggs	caproate (6:0)	Medium Chain Fatty Acid	0.9855	(0.9724,0.9987)	0.0364
	X-16982	unknown	1.0130	(1.0008,1.0254)	0.0406
Fish	3-carboxy-4-methyl-5-propyl-2-furanpropanoate (CMPF)	Fatty Acid, Dicarboxylate	1.0136	(1.0073,1.02)	1e-04
	erucate (22:1n9)	Long Chain Fatty Acid	1.0088	(1.0021,1.0155)	0.0158
	eicosenoate (20:1)	Long Chain Fatty Acid	1.0044	(1.0005,1.0082)	0.0349
	1-docosahexaenoyl-GPC (22:6)*	Lysolipid	1.0087	(1.0026,1.0147)	0.0065

Supplementary data

Food group	Metabolite	Subpathway	β-Estimator	Confidence interval (CI)	p.value
	1-eicosapentaenoyl-GPC (20:5)*	Lysolipid	1.0053	(1.0016,1.0091)	0.0059
	1-docosahexaenoyl-GPE (22:6)*	Lysolipid	1.0039	(1.0011,1.0067)	0.0095
	eicosapentaenoate (EPA 20:5n3)	Polyunsaturated Fatty Acid (n3 and n6)	1.0062	(1.0031,1.0093)	4e-04
	docosahexaenoate (DHA 22:6n3)	Polyunsaturated Fatty Acid (n3 and n6)	1.0062	(1.0031,1.0093)	5e-04
	docosapentaenoate (n3DPA 22:5n3)	Polyunsaturated Fatty Acid (n3 and n6)	1.0038	(1.0005,1.0071)	0.0325
	linolenate [alpha or gamma; (18:3n3or6)]	Polyunsaturated Fatty Acid (n3 and n6)	1.0040	(1.0008,1.0072)	0.017
	X-02269	unknown	1.0117	(1.0069,1.0166)	<.0001
Fruits	X-11315	unknown	1.0004	(1,1.0008)	0.0345
	X-17145	unknown	1.0011	(1.0001,1.0021)	0.04
Margarines	X-11261	unknown	1.0088	(1.0037,1.0139)	9e-04
	X-11478	unknown	1.0105	(1.0011,1.0199)	0.031
	X-11521	unknown	1.0099	(1.0023,1.0177)	0.0123
Milk	2-aminoheptanoate	Fatty Acid, Amino	0.9987	(0.9977,0.9997)	0.0148
	X-21365	unknown	1.0004	(1.0001,1.0006)	0.0079
Nuts and seeds	indolepropionate	Tryptophan Metabolism	1.0265	(1.0009,1.0528)	0.0445
Other cereals	X-09789	unknown	1.0195	(1.0064,1.0328)	0.004
Potatoes	4-hydroxyphenylpyruvate	Phenylalanine and Tyrosine Metabolism	0.9958	(0.9919,0.9998)	0.0464
Root vegetables	oxalate (ethanedioate)	Ascorbate and Aldarate Metabolism	1.0031	(1.0002,1.0061)	0.0401
	X-17259	unknown	1.0115	(1.0008,1.0223)	0.0373
Spirits	p-cresol sulfate	Phenylalanine and Tyrosine Metabolism	0.9784	(0.9578,0.9995)	0.0492
Sugar	erucate (22:1n9)	Long Chain Fatty Acid	0.9934	(0.987,0.9997)	0.0471
	1-pentadecanoyl-GPC (15:0)*	Lysolipid	1.0070	(1.0003,1.0138)	0.0446

Supplementary data

Table S6: Food-metabolite associations in the identification analysis and their corresponding back transformed estimator and confidence interval found in the test dataset

Food group	Metabolite	Estimator	Confidence Interval (CI)	p-Value*
Coffee	paraxanthine[1]	1.0008	(1.0004, 1.0011)	0.0346
	X-14473[2]	1.0013	(1.0007, 1.0019)	0.0473
Fish	eicosapentaenoate (EPA_20:5n3)[1]	1.0068	(1.0039, 1.0098)	0.0445
	X-02269[1]	1.0112	(1.0067, 1.0158)	0.0054

[1] found in men; found in [2]women; *adjusted for multiple testing according to Bonferroni correction

Table S7: Food groups used in the present analysis

**Number**	**Food group**	**Corresponding food items**
1	Potatoes	Potatoes
2	Leafy vegetables	Leafy vegetables
3	Fruiting vegetables	Fruiting vegetables
4	Root vegetables	Root vegetables
5	Cabbages	Cabbages
6	Other vegetables	Mushrooms
		Pods vegetables
		Onions
		Stalk vegetables
		Salad
7	Legumes	Legumes
8	Fruits	Fruits
9	Nuts and seeds	Nuts and seeds
10	Other fruits	Mixed Fruits
11	Milk	Milk
12	Dairy products	Dairy drinks
		Yogurt
		Crud
		Cream desserts pudding
		Creme sour creme
		Coffee Cream Coffee Whitener
13	Cheese	Cheese
14	Pasta, rice	Noodles rice other Grains
15	Bread	Bread
		Crispbread rusk
16	Other cereals	Flour flakes starch
		Breakfast cereals
		Dough
17	Red meat	Unclassified Meat Meat products
		Beef
		Pork

Supplementary data

Number	Food group	Corresponding food items
18	Poultry	Unclassified and other chicken
		Chicken
19	Processed meat	Sausages
20	Offals	Offal
21	Fish	Fish
		Fish products
22	Eggs	Egg
23	Vegetable oil	Vegetable oil
24	Margarines	Margarine
25	Butter	Butter
26	Other fats	Unclassified fat
		Frying fat
		Animal fat
27	Sugar	Sugar honey jam
		Sweets without chocolate
		Syrup
		Ice cream
28	Chocolate	Chocolate sweets
29	Cake, cookies	Cakes pastries
		Biscuits
30	Fruit and vegetable juice	Fruit and vegetable juices
31	Soft drinks	Lemonade Coke etc
32	Coffee	Coffee
33	Tea	Tea
		Herbal tea
34	Other non-alcoholic drinks	Unclassified non-alcoholic drinks
		Water
35	Wine	Wine
36	Beer, Cidre	Beer Cidre
37	Spirits	Spirits
38	Other alcoholic beverages	Dessert wine
		Aniseed drinks
		Liqueur
		Cocktails punch
39	Sauces	Unclassified sauce
		Tomato sauce
		Dressings

Supplementary data

Number	Food group	Corresponding food items
		Mayonnaise
		Dessert sauce
40	Soups	Soup
		Bouillon
41	Miscellaneous	Spices herbs
		Snacks
		Soy products
		Sweeteners

Table S8: Annotation and metabolite descriptions

Cf. supplement: Excel file

Table S9: Applied search term

Field	Search term
Exposure	("Diet"[Mesh] OR "Diet"[All Fields] OR "Food"[Mesh] OR "Food"[All Fields] "Dietary intake"[All Fields] OR "Food intake"[All Fields])
Outcome	("Biomarkers"[Mesh] OR "biomarkers"[All Fields] OR "metabolite"[All Fields] OR "metabolites"[All Fields] OR "metabolite profile"[All Fields] OR "metabolomics signature"[All Fields] OR "Metabolomics"[Mesh] OR "Metabolomics"[All Fields] OR "Metabolome"[Mesh] OR "Metabolome"[All Fields])
Biospecimen	("Blood"[Mesh] OR "Blood"[All Fields] OR "serum"[All Fields] OR "plasma"[All Fields])
Study design	("Cohort Studies"[Mesh] OR "Cohort Studies"[All fields] OR "Case-Control Studies"[Mesh] OR "Case-Control Studies"[All fields] OR "Cross-Sectional Studies"[Mesh] OR "Cross-Sectional Studies"[All fields] OR "Prospective Studies"[Mesh] OR "Prospective Studies"[All fields] OR "Retrospective Studies"[Mesh] "Retrospective Studies"[All fields] OR "Epidemiologic Studies"[Mesh] OR "Epidemiologic Studies"[All Fields] OR "observational"[All Fields]) NOT ("in vitro techniques"[mh:noexp]) NOT ("animals"[mh:noexp])

Supplementary data
